# Family Perfectionism among Russian College Students

**DOI:** 10.11621/pir.2022.0303

**Published:** 2022-09-15

**Authors:** Emily E. Camp, Marina S. Sheveleva, Tatiana M. Permyakova, Kenneth T. Wang

**Affiliations:** a University of Nebraska-Lincoln, USA; b HSE University, Russia; c Fuller Theological Seminary, USA

**Keywords:** Perfectionism, family, Russian, college students, achievement, psychometric evaluation

## Abstract

**Background:**

Research documenting the consequences of perfectionism on psychopathology and academic achievement across diverse cultures proliferates. This paper situates the multidimensional model of perfectionism and the role of family perfectionism within a Russian context.

**Objective:**

The main purposes are to investigate the psychometric properties of the Family Almost Perfect Scale (FAPS) among Russian college students and to explore whether the different types of perfectionistic families found in past studies are replicated in the sample. The impact of both personal and family aspects of perfectionism on psychological and academic outcomes is investigated.

**Design:**

The psychometric properties of a Russian family perfectionism measure were examined using 169 students (50 men, 119 women), recruited at a national university in Perm, Russia. Their overall average age was 19.60 (*SD* = 0.63), ranging from 18 to 23 (Men: *M* = 19.72, *SD* = 0.76; Women: *M* = 19.55, *SD* = 0.56).

**Results:**

Results indicated that the adjusted 15-item Russian Family Almost Perfect Scale (FAPS) yielded adequate factor structure, construct validity, and internal consistency reliability. The distinctively adaptive and maladaptive natures of the Family Standards and Family Discrepancy subscales were supported through correlations with psychological distress measures, as well as the three different types of perfectionistic families that were replicated through cluster analyses. The adaptive, maladaptive, and non-perfectionistic families mirrored the groups found in past studies. In comparing individuals of various family types, those from maladaptive perfectionistic families reported higher levels of depressive mood and anxiety than those from adaptive perfectionistic families.

**Conclusion:**

Findings implicate the relevancy of this construct to college students’ psychological well-being. The Russian FAPS could be used in future research to further explore perceived family perfectionism among Russian-speaking populations.

## Introduction

Recent decades have witnessed an explosion in research documenting the deleterious consequences of perfectionism on psychopathology and academic achievement across racially and ethnically diverse cultures and countries (Methikalam et al., 2015; Ortega et al., 2014; Wang et al., 2012; Wang et al., 2018). Perfectionism is defined as a tendency to set high standards for performance with unremitting efforts to attain these standards, where one may measure their own self-worth in terms of their accomplishments, often resulting in critical self-evaluation (Shafran et al., 2002; Stoeber & Otto, 2006; Wang et al., 2019). Research suggests that the development of perfectionism relates to experiences within one’s family, particularly the parental influence (Walton et al., 2020). Many studies have linked perfectionism to a range of psychological concerns including depression, anxiety, obsessive-compulsive disorder (OCD), and eating disorders (Hu et al., 2019; Jun et al., 2022; Limburg et al., 2017; Methikalan et al., 2015), as well as negative public health outcomes like suicide (Smith et al., 2018). Additionally, perfectionism has been negatively linked with indicators of academic achievement such as Grade Point Average (GPA), self-reported academic satisfaction, and academic-related anxiety (Filippello et al., 2019; Osenk et al., 2020; Wang et al., 2018; Yang et al., 2019), impacting elementary to college age students. As a result of the familial development of perfectionism and long-lasting deleterious consequences, research on perfectionism within families is sorely needed to inform education, prevention, and intervention efforts. In this article, we provide an overview of the multidimensional model of perfectionism and situate the role of family perfectionism within a Russian context. This is followed by an evaluation of the psychometrics of the Russian version of the Family Almost Perfect Scale and an examination of the personal and familial aspects of perfectionism among Russian students. Finally, we illuminate the importance of this measure for cross-cultural perfectionism theory and describe the practical value for psychologists, interventionists, and educators working with Russian students and families.

The Hamacheck theory of perfectionism (1978) provides a conceptual framework for understanding the antecedents, characteristics, and behavioral symptoms of a multidimensional model of perfectionism. In terms of antecedents, the development of perfectionism stems from family environments with parent-set performance standards for children and related conditional or unconditional approval. Family environments foster the development of adaptive and maladaptive forms of perfectionism, termed “normal perfectionism” and “neurotic perfectionism,” respectively (Filippello et al., 2019; Hamachek, 1978; Wang et al., 2018). Normal perfectionists set realistic standards for themselves and enjoy striving towards such standards; neurotic perfectionists, on the other hand, set unrealistic standards of achievement and are critical of the discrepancy between their actual performance and desired level of achievement resulting in dissatisfaction, self-criticism, and suffering (Hamachek, 1978; Stoeber, 2006). Non-perfectionists show low levels of perfectionism as they are less prone to strive towards high standards. The dual nature of perfectionism was hypothesized to differentially impact domains of functioning related to health and academia, underscoring the need to understand perfectionism as having adaptive and maladaptive effects (Stoeber et al., 2020).

Over thirty years of factor analysis research supports the multidimensional model of perfectionism as a construct with adaptive and maladaptive components that differentially impact individual psychological health and achievement (Frost et al., 1990; Herman et al., 2011; Hewitt & Flett, 1991; Slaney et al., 2001; Vacca et al., 2020). The components are termed perfectionistic striving (PS) and perfectionistic concerns (PC). PS refers to demanding perfection of oneself and setting high personal standards, while PC refers to the tendency to be overly focused on imperfections, mistakes, criticism, and perceived discrepancies between ideal and actual achievements (Stoeber & Otto, 2006; Wang et al., 2016). While PC is consistently positively associated with maladaptive outcomes such as negative affect and academic difficulties, PS often positively relates to adaptive outcomes such as positive affect and academic achievement (Farag, 2020; Stoeber et al., 2020).

The multidimensional perfectionistic model is reflected in a range of scales. Notable examples include Frost’s Multidimensional Perfectionism Scale (FMPS; Frost et al., 1990) and Hewitt and Flett’s Multidimensional Perfectionism Scale (HFMPS; Hewitt & Flett, 1991). However, following critique of both scales in the context of family perfectionism (Wang, 2010), the development of the scale for measuring family perfectionism proceeded from a different scale. Namely, The Almost Perfect Scale-Revised (APS-R; Slaney et al., 2001). It has a track record for measuring perfectionism in the international context (Wang et al., 2012) and contains three subscales. The Standards and Order subscales measure the positive aspects of perfectionism. The Discrepancy subscale assesses the negative aspect of perfectionism, or an individuals’ tendency to perceive a gap between their standards and actual performance. Based on the combination of these APS-R subscale scores, participants have been classified into adaptive, maladaptive, and non-perfectionist categories (Grzegorek et al., 2004; Rice & Slaney, 2002). Rice and colleagues (2014) refined the APS-R to measure the major dimensions of perfectionism as Standard (high performance expectations) and Discrepancy (self-critical performance evaluations), reflecting the PS and PC domains via the Short Almost Perfect Scale (SAPS; Rice et al., 2014). The SAPS demonstrates good psychometric features as a brief and strong measure of major perfectionism factors and types (e.g., non-perfectionists and adaptive and maladaptive perfectionists; Rice et al., 2014). An accumulation of evidence suggests that these types of perfectionists are differentially associated with important outcomes such as psychological distress and academic achievement ( Grzegorek et al., 2004; Mobley et al., 2005; Ocampo et al., 2020; Osenk et al., 2020; Rice & Ashby, 2007; Rice & Dellwo, 2002; Rice & Slaney, 2002).

The developmental nature of perfectionism within families suggests that several variables are related to the transmission of perfection. An important variable concerns the level of discrepancy children perceive between their family’s high expectations and their actual performance. When a child perceives a high levels of discrepancy between what is expected by the family and the child’s own perception of their performance, distress may occur and manifest in psychological difficulties such as depression, anxiety, and suicidal ideation (Rasmussen & Troilo, 2016). When individuals perceive discrepancies between family expectations (e.g., “I’m not meeting X expectation in my family”), it is significantly and positively associated with individual’s personal experience of discrepancy (e.g., “I’m not meeting X expectation I have for myself either”) (Rasmussen & Troilo, 2016; Wang et al., 2013). In other words, difficulty meeting the high expectations of one’s family may result or is reflected in difficulty meeting one’s own expectations, highlighting the importance of considering family variables when studying individual perfectionism outcomes.

The Family Almost Perfect Scale (FAPS; Wang, 2010) was developed to expand the personal adaptive and maladaptive perfectionist literature to include perceived family perfectionism. The FAPS, consisting of three subscales – Family Standards, Family Order, and Family Discrepancy, mirrors the multidimensional framework of perfectionism literature by differentiating parallel types of perfectionistic families (e.g., adaptive, maladaptive, and non-perfectionist). Though the FAPS was initially developed to study perfectionism among populations with strong family values in a collectivist context, the model has been validated among various culturally and ethnically diverse groups such as Asian and European Americans (Carrera & Wei, 2017; Methikalam et al., 2015; Wei et al., 2013), Latino/a Americans (Ortega et al., 2014), Asian Indians (Wang et al., 2012), Chinese students (Deng et al., 2012), Italians (Filippello et al., 2019), and Greek students (Diana & Spyridon, 2018). With growing interest in understanding developmental antecedents, moderators, and mediators of perfectionistic outcomes, the FAPS has been translated into multiple languages including Chinese, Greek, Italian, Korean, Lithuanian, and Russian. Given that the FAPS demonstrates adequate psychometric properties and cross-cultural construct validity, it is a highly relevant measure for examining other culturally and ethnically diverse populations with demonstrated perfectionistic tendencies and outcomes.

Previous studies indicate that Russian individuals demonstrate perfectionistic tendencies and may be at a unique risk for negative affective and academic outcomes. The majority of studies highlight the effects of maladaptive perfectionism on psychological well-being (Wang et al., 2016) and have found a positive relationship between maladaptive perfectionism and suicide attempts (Sokolova & Tsygankova, 2011), hostility and stress (Kholmogorova et al., 2009), depression and anxiety (Garanyan & Yudeeva, 2009), foreign language learning and classroom anxiety (Wang et al., 2018), and imposter syndrome (Wang et al., 2019). Yasnaya and colleagues (2011) translated the Almost Perfect Scale-Revised and found that the factor structure of Russian translated scales coincided with the original scale. More recently, Wang and colleagues (2016) translated and evaluated the psychometric properties of a Russian version of the Short Almost Perfect Scale (SAPS; Rice et al., 2014) and found that the factor structure was supported and construct validity was established through its relationship with anxiety and depression scores among Russian college students. These studies provide evidence for deleterious outcomes associated with maladaptive perfectionism. Yet, further research is needed to understand the antecedents of perfectionism and to better inform intervention efforts that may reduce negative outcomes for students.

In line with the developmental understanding of perfectionism, research indicates that Russian students with high levels of perfectionism may come from families with high expectations (Evenko, 2014; Volikova, 2012). A few Russian studies have examined how parental perfectionism impacts children. Kovalenko (2012) argues that parents’ perfectionist attitudes influence the psychological health of a child from as early as elementary school. Larskikh & Shiryaev (2016) found correlations between family perfectionism and personal perfectionism in students. Namely, students from families with high family perfectionism demonstrated perfectionism, and female students faced significantly higher levels of family perfectionism than male students (Larskikh & Shiryaev, 2016). Volikova (2012) found an association between perfectionism and emotional problems in children and adolescents, stressing that parental perfectionism has a negative influence on excessive criticism, pressure, and rejection. In a sample of 68 teenagers and their mothers, Evenko (2014) found higher levels of perfectionism among authoritarian parents (12%) and lower levels among liberal parents (73%). Further, Tarkhanova (2014) found that family perfectionism is closely linked to high levels of appearance perfectionism and emotional problems among young females. According to Yakimova and Kravtsova’s (2015) study, family perfectionism can be characterized by patriarchal attitudes that require strict fulfillment of home duties. Andreeva (2015) also found that patriarchal attitudes are associated with higher family perfectionism and traits like high expectations, controlling parenting styles, and a strong concern for self-image. These studies suggest that family perfectionism is related to high expectations and controlling behaviors that may lead to low self-esteem and psychological distress in children.

Little is known about how varied types of Russian perfectionistic families (e.g., non-perfectionistic, maladaptive perfectionistic, and adaptive perfectionistic) are associated with varied perfectionism styles in young people and the related maladaptive and adaptive outcomes (Evenko, 2014). In other words, research is limited on how non-perfectionistic, maladaptive, and adaptive family perfectionism relates to non-perfectionistic, maladaptive, and adaptive perfectionism among the Russian youth (Andreeva, 2015). Thus, the main purposes of this study are 1) to investigate the psychometric properties of the Family Almost Perfect Scale (FAPS) among Russian college students, as this scale in unavailable in Russia; 2) to explore whether the different types of perfectionistic families found in past studies are replicated in this sample; 3) to examine the impact of both personal and family aspects of perfectionism among Russian students on psychological and academic outcomes. To accomplish these goals, a cross-sectional survey was designed. First, a confirmatory factor analysis to examine the factor structure of the Russian FAPS. Second, establishing construct validity via a comparison of the correlations between FAPS subscales scores with SAPS subscales and those of other variables. To this end, we hypothesized that family discrepancy is positively associated with depression symptoms and anxiety. Third, a cluster analysis to classify individuals into different types of perfectionistic families. These groups of perfectionistic families are to be compared on psychological distress variables to establish their adaptive and maladaptive natures. Based on previous cluster analysis studies (Wang et al., 2016), we believe that there would be three groups of perfectionistic families: adaptive, maladaptive, and non-perfectionists. We also hypothesized that students from adaptive perfectionistic families would report better psychological outcomes than those from maladaptive perfectionistic families. We also compared the groups on their academic performances.

## Methods

### Participants

Participants were 169 college students (50 men, 119 women) at a national university in Russia, recruited as part of a larger study. Their overall average age was 19.60 (*SD* = 0.63), ranging from 18 to 23 (Men: *M* = 19.72, *SD* = 0.76; Women: *M* = 19.55, *SD* = 0.56). These students majored in various fields: 32% in management, 27% in economics, 17 % in business informatics, 12% in law, and 11% in program engineering. After indicating active consent and being informed that their participation in the research was voluntary, participants included in the present study chose to stay after class to complete this pen-and-paper questionnaire. The research survey was presented in Russian and took 20–30 minutes to complete. No financial compensation nor additional incentives were provided for participation. The study procedures complied with the research ethical code of the institution in which the participants were recruited.

### Procedure

#### Family Almost Perfect Scale (FAPS; Wang, 2010).

The FAPS was used to measure the participants’ level of perceived perfectionism within their family. The FAPS consists of three subscales — Standards (6 items), Order (4 items) and Discrepancy (7 items). The Family Standards subscale measures the perceived degree of high standards for achievement and performance set by one’s family; a sample item states, “My family sets very high standards for me.” The Family Order subscale measures the perceived family preference for neatness and orderliness; a sample item states, “My family expects me to be an orderly person.” The Family Discrepancy subscale measures the perception of falling short of family expectations for performance; a sample item states, “Doing my best never seems to be enough for my family.” Participants rated each item on a seven-point Likert scale: 1 (strongly disagree) to 7 (strongly agree). The FAPS was translated into Russian on the basis of Brislin’s (1980) three-step back-translation guidelines. Cronbach’s alpha coefficients for the FAPS subscale scores ranged from 0.78 to 0.94 in samples of Asian Americans and European Americans (Wang, 2010). In the current study, Cronbach’s alpha for Family Standards, Family Order, and Family Discrepancy scores was 0.84, 0.78, and 0.89, respectively.

#### Short Almost Perfect Scale (SAPS; Rice et al., 2014).

The SAPS was used to measure participants’ own perfectionism level. The SAPS is an 8-item brief version of the Almost Perfect Scale-Revised (Slaney et al., 2001), and consists of two subscales — Standards (4 items) and Discrepancy (4 items). The Standards subscale measures the level of one’s striving for perfection by setting extremely high expectations. A sample Standards item states, “I have a strong need to strive for excellence.” The Discrepancy subscale measures one’s tendency of feeling inadequate by a constant focus on a perceived gap between one’s standards and performance. A sample Discrepancy item states, “Doing my best never seems to be enough.” Participants rated each item on a seven-point Likert scale: 1 (strongly disagree) to 7 (strongly agree). In a prior study, the SAPS was translated into Russian on the basis of Brislin’s (1980) three-step back-translation guidelines, and Cronbach’s alpha was 0.79 for Standards and 0.78 for Discrepancy in a different Russian college student sample (Wang et al., 2016). In the current study, Cronbach’s alpha for Standards and Discrepancy scores were 0.81 and 0.80, respectively.

#### Depression Anxiety Stress Scale-21 (DASS-21; Lovibond & Lovibond, 1995).

The DASS was used to measure psychological distress through three subscales — Depression (7 items), Anxiety (7 items), and Stress (7 items). A sample Depression item states, “I couldn’t seem to experience any positive feeling at all.” A sample Anxiety item states, “I felt scared without any good reason.” A sample Stress item states, “I tended to overreact to situations.” The DASS is rated on a four-point Likert scale: 0 (did not apply to me at all) to 3 (applied to me very much, or most of the time). We used a Russian version of the DASS obtained from the DASS website (www.psy.unsw.edu.au/dass/). Cronbach’s alpha for the Depression, Anxiety, and Stress subscale scores ranged from 0.77 to 0.87 in a previous sample of Russian students (Wang et al., 2016). In the current study, Cronbach’s alpha for the Depression, Anxiety, and Stress scores was 0.84, 0.83, and 0.84, respectively.

#### Grade Point Average

Participants self-reported their academic grade average. The grade scaling at the institution of their study was as follows: 8-10 = excellent (A equivalent), 6–7 = good (B equivalent), 4–5 = satisfactory (C equivalent), below 4 = unsatisfactory (D equivalent, or fail).

#### Data Analytic Plan

Missing data was examined, and participants (*n* = 3) with any missing data were not included in the study. First, a confirmatory factor analysis (CFA) was conducted to examine the factor structure of the Russian FAPS. The construct validity and reliability of the Russian FAPS was then investigated. Finally, cluster analysis was used to identify different types of perfectionistic families and the cluster types on psychological distress and academic performances were compared using ANOVA. CFA was conducted by Mplus and all other analyses by SPSS.

## Results

We first examined the factor structure of the Russian FAPS. As the FAPS is an established measure with psychometric evaluations completed on various populations, confirmatory factor analyses (CFA) were carried out first to examine its factor structure with this Russian sample. Normality of the items was considered first; skewness ranged from –1.31 to 1.28 and kurtosis ranged from –0.82 to 1.99. Due to the slight deviation from normality, we used Robust Maximum Likelihood estimation for CFA. The following indices were used to assess model fit: comparative fit index (CFI), the standardized root-mean-square residual (SRMR), and the root-mean-square error of approximation (RMSEA). CFI above 0.90 (Byrne, 1998) or 0.95 (Hu & Bentler, 1999) indicates an acceptable data to model fit. Lower RMSEA values indicate better fit, values less than 0.05 suggest a close fit, between 0.05 and 0.08 a fair fit, between 0.08 and 0.10 a mediocre fit, and over 0.10 a poor fit (MacCallum et al., 1996). Similarly, lower SRMR values suggest better fit, and a value below 0.08 is desired (Hu & Bentler, 1999).

**Figure 1.**
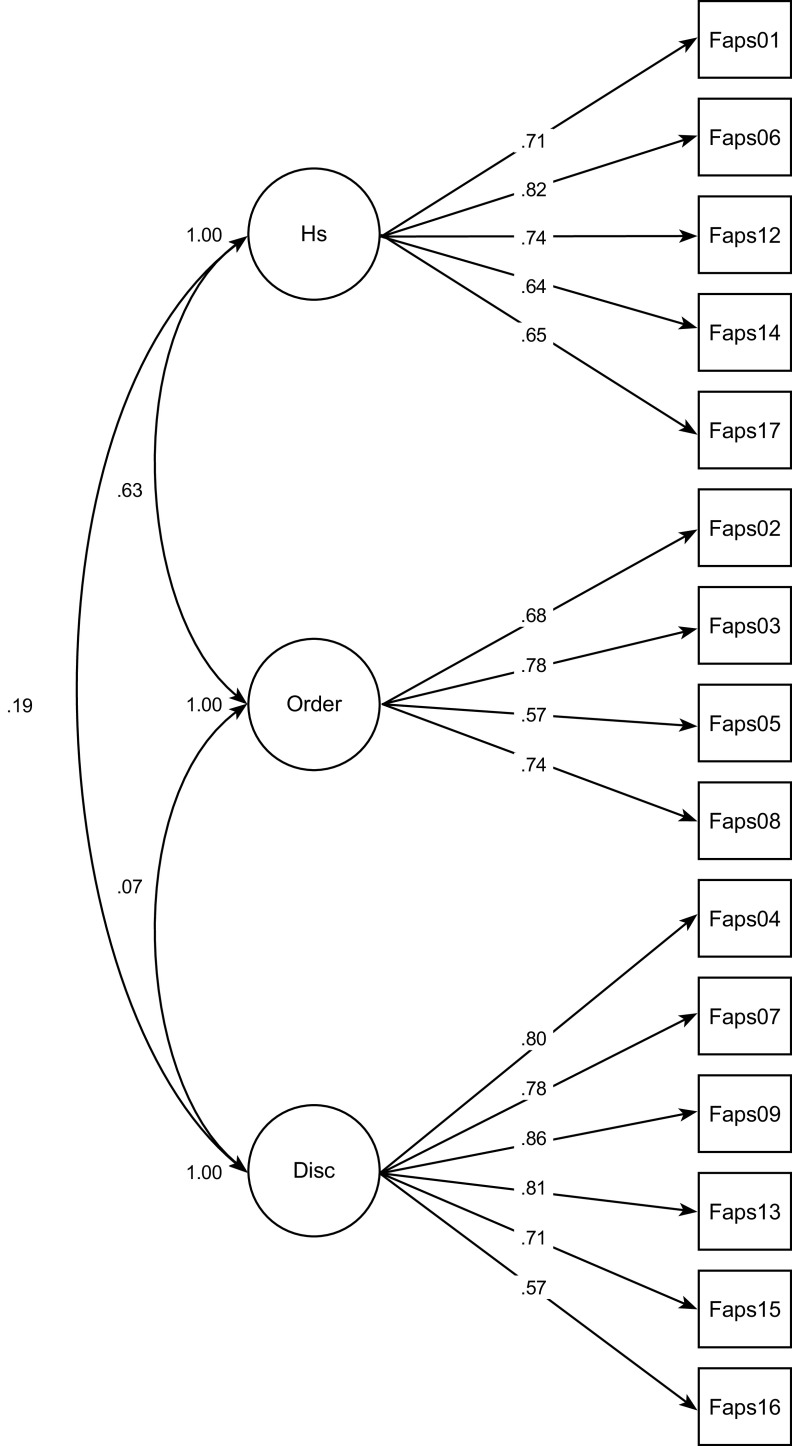
Confirmatory Factor Analysis (CFA) Diagram

The initial CFA model for FAPS constrained the 6 Family Standards items, 7 Family Discrepancy items, and 4 Family Order items to load onto their corresponding factors. In this oblique model using Robust Maximum Likelihood estimation and Geomin rotation, the factors were permitted to correlate with one another. The model yielded the following fit statistics: MLRχ^2^(116, *N* = 169) = 209.96, *p* < .001, CFI = .856, SRMR = .092, RMSEA = .094. These results were less than ideal. Thus, we made step-by-step modifications based on the modification indices. Two items (#10 “My family sets very high standards for me.” and #11 “Nothing short of perfect is acceptable in my family.”) were removed due to substantial cross loading on a second factor. The adjusted FAPS for this Russian sample included 15 items and yielded the following model fit statistics: MLRχ^2^(87, *N* = 169) = 182.52, *p* < .001, CFI = .901, SRMR = .064, RMSEA = 0.081, indicating adequate fit. Standardized factor loadings ranged from .64 to .82 for Family Standards items, 0.57 to 0.78 for Family Order items, and .57 to .87 for Family Discrepancy items. The factor correlations were as expected. Family Standards and Family Order were strongly correlated with each other (*r* = .63), as they were both viewed as positive aspects of perfectionism. Family Discrepancy was weakly correlated with Family Standards (*r* = 0.19) and minimally correlated with Family Order (*r* = .07). See *Figure 1* for CFA diagram with each factor loading.

*Table 1* presents means, standard deviations, reliability coefficients, and inter-correlations. All measures used to assess study variables had adequate internal consistencies, with Cronbach’s alphas ranging from 0.72 to 0.89. Overall, the personal perfectionism and family perfectionism dimensions correlated in the expected directions. Pearson’s correlation coefficients between the APS-R and FAPS corresponding subscale scores were 0.47 for Standards and 0.42 for Discrepancy, providing evidence for convergent validity. In contrast, correlation coefficients across non-corresponding subscales of the APS-R and FAPS (i.e., FAPS Standard with APS-R Discrepancy and FAPS Discrepancy with APS-R Standards) were minimal, ranging from -0.04 to -0.09, providing support for divergent validity. Family Discrepancy was positively correlated with depressive mood, anxiety, and stress, whereas Family Standards was not significantly correlated with any of these three psychological distress variables. These trends provide support for the adaptive and maladaptive nature of Family Standards and Family Discrepancy, respectively. However, there were some interesting differences between how family and personal standards correlated with other variables. For example, Family Standards were negatively but weakly correlated with GPA, whereas personal Standards were positively but weakly correlated with GPA.

**Table 1:** Means, Standards Deviations, Correlations, and Alphas of Study Variables (N = 169)

Variables	1	2	3	4	5	6	7	8
1. Family Standard s								
2. Family Order	0.51***							
3. Family Discrepancy	0.19*	0.06						
4. Personal Standards	0.47***	0.27***	–0.09					
5. Personal Discrepancy	–0.04	–0.02	0.42***	0.00				
6. Depressive Mood	–0.12	–0.17*	0.43***	–0.25**	0.44***			
7. Anxiety	–0.02	–0.06	0.29***	–0.12	0.28***	0.63***		
8. Stress	0.06	0.02	0.30***	–0.08	0.29***	0.59***	0.71***	
9. Grade Point Average	–0.17*	–0.05	–0.10	0.18*	0.06	–0.07	–0.11	–0.06
**Mean**	25.67	20.69	15.75	21.06	15.66	6.33	5.05	9.33
**S.D.**	5.07	4.21	6.23	4.24	4.81	4.66	4.38	4.79
**alpha**	0.84	0.78	0.89	0.81	0.80	0.84	0.83	0.84

Note. * p < 0.05, ** p < 0.01, *** p < 0.001.

Cluster analysis was used to classify participants into different family types based on their FAPS Family Standards and Family Discrepancy scores. Following Wang’s (2010) two-step cluster analyses procedure, both hierarchical and nonhierarchical analyses were conducted. As a first step, hierarchical cluster analysis was performed with standardized Family Standards and Family Discrepancy scores using Ward’s linkage method with the squared Euclidian distance measure. A relatively large increase in the agglomeration coefficient (40%) occurred when the solution decreased from three to two clusters, indicating that the two clusters joining at that particular step resulted in a less homogeneous joint cluster (Hair et al., 2000). Thus, the three-cluster solution was used for the second step. This involved a nonhierarchical k-means cluster analysis using the standardized means of each cluster’s Family Standards and Family Discrepancy scores as starting values.

Participants were classified into three groups — adaptive perfectionistic families (high-Family Standards and low-Family Discrepancy; *n* = 75), maladaptive perfectionistic families (high-Family Standards and high-Family Discrepancy; *n* = 41), and non-perfectionistic families (low-Family Standards and low-Family Discrepancy; *n* = 53). To examine the influence of gender, a chi-squared test was conducted. No significant gender distribution differences were found across the three family types [χ^2^ = (2, *N* = 169) = 3.16, *p* = 0.21]. The three perfectionistic family types were then compared on study variables (see *Table 2*). Participants in the maladaptive perfectionistic family group reported significantly higher levels of depressive mood and anxiety symptoms than the adaptive perfectionistic family group. Th ere were no significant differences between the two perfectionistic family groups regarding stress and GPA.

**Table 2:** Means and Standard Deviations by Perfectionistic Family Groups

	Adaptive Perfectionistic Families *n* = 75		Maladaptive Perfectionistic Families *n* = 41		Non-Perfectionistic Families *n* = 53			
Subscale	M	SD	M	SD	M	SD	F	η^2^
Family Standards	28.84^a^	2.92	27.02^b^	3.36	20.15^c^	3.88	108.57	0.57
Family Order	22.21^a^	3.66	20.76^a^	3.59	18.49^b^	4.48	14.01	0.14
Family Discrepancy	13.41^a^	3.40	24.05^b^	5.36	12.64^a^	3.87	111.65	0.57
Personal Standards	22.60^a^	3.66	20.44^b^	3.78	19.36^b^	4.63	10.77	0.12
Personal Discrepancy	14.36^a^	4.40	18.07^b^	5.08	15.64^a^	4.51	8.61	0.09
Depressive Mood	4.95^a^	3.74	8.76^b^	5.54	6.40^a^	4.37	9.80	0.11
Anxiety	4.15^a^	3.73	6.32^b^	5.20	5.36^ab^	4.34	3.55	0.04
Stress	8.96^ab^	4.60	10.85^a^	4.85	8.66^b^	4.84	2.88	0.03
Grade Point Average	7.04	1.03	6.99	1.17	7.35	0.89	1.86	0.02

*Note. All univariate ANOVA F tests were significant at p < 0.05, except for Stress (p = 0.059) and Grade Point Average (p = 0.157). F tests were based on df = 2,166. Values with different superscripts indicate significant within-row differences between the clusters using Tukey HSD post hoc comparisons, significant at p < 0.05.*

## Discussion

The aim of the current study was to determine the psychometric properties of the Russian FAPS and to examine the impact of both personal and familial aspects of perfectionism on psychological and academic outcomes. Although several perfectionism studies have provided evidence of the impact of family perfectionism on students (Filippello et al., 2019; Methikalam et al., 2015; Ortega et al., 2014; Wang, 2012; Wang et al., 2012), the current paper is the first to examine the psychometric properties of a family perfectionism measure and its relationship to psychological and academic outcomes among a Russian population. Results indicated that the adjusted 15-item Russian FAPS yielded adequate factor structure, construct validity, and internal consistency reliability. The distinctively adaptive and maladaptive nature of the Standard and Discrepancy subscales were also supported through the correlations with psychological distress measures, as well as the three different types of perfectionistic families that were replicated through cluster analyses. Namely, the adaptive, maladaptive, and non-perfectionist families mirrored the groups found among Asian American (Wang, 2010) and Latinx (Ortega et al., 2014) samples.

In line with previous research (Ortega et al., 2014; Wang, 2010), when comparing individuals of various family types through ANOVA, those from maladaptive perfectionistic families reported higher levels of depressive mood and anxiety than those from adaptive perfectionistic families. In other words, families that expect high standards (e.g., adaptive perfectionistic families) do not necessarily facilitate psychological distress for students. However, our findings indicate that students from maladaptive perfectionistic families experience more psychological stress. Based on participant perceptions, these students’ families not only expect high performance, but also constantly indicate that the students’ performances are not good enough. Students from maladaptive perfectionistic families also reported higher levels of depressive mood and stress compared to those from non-perfectionistic families. Th ere are several potential explanations for this finding. In line with previous research, students who experience discrepancy between the family’s standards and their ability to meet those standards (e.g., maladaptive perfectionists) may experience feelings of shame and imposter syndrome that could trigger their depressive and anxious experiences (Hu et al., 2019; Wang et al., 2018; Wang et al., 2019; Wei et al., 2020; Yoon & Lau, 2008). It may also be that maladaptive perfectionist students strive to excel and seek high personal and familial standards more than non-perfectionists. As a result, they might be more heavily impacted by differences between their standards and their ability to meet those standards, resulting in graver mental health difficulties than non-perfectionistic students (Nelsen et al., 2021).

Despite experiencing more psychological distress, the academic performance of the students from maladaptive perfectionistic families, measured by GPA, did not differ from those in the two other groups. Therefore, the consistent feeling of not measuring up to family expectations was not due to poorer performance, at least in the academic domain. Only a few studies have highlighted links between maladaptive family perfectionism and poor academic performance (Haddadi & Tamannaeifar, 2022; Kawamura et al., 2002; Yang et al., 2016). In fact, one explanation for this non-significant finding might be that family perfectionism is more tied to academic distress and worry than academic performance (Jun et al., 2022). Academic distress (e.g., feelings of motivation and worries about academic ability) may differ from academic performance, such that students who feel highly distressed in school may still perform objectively well (Akgun & Ciarrochi, 2003; Bedewy & Gabriel, 2015). Thus, future studies should examine the impact of family perfectionistic types on academic distress and performance, as well as factors which may mediate the relationship between the two variables. Nevertheless, one’s perception of never being good enough in their family’s eyes is an important issue for individuals’ psychological well-being that deserves more attention and further examination.

This study was an important first step in providing support for the relevance of family perfectionism in the development of individual perfectionism, especially among students within Russian culture. As such, the findings of the study may hold important practical implications for instructors, clinicians, counselors, and advocates working with Russian individuals and families. First, the study suggests that individual and intrinsic perfectionism may stem from familial influences, such as how one perceives perfectionistic expectations and criticisms from their family. To tend to the well-being of students and individuals struggling with perfectionism, providers should consider how an individual’s feelings of inadequacy are influenced by familial factors. Thus, it may be helpful to explore the source of an individual’s sense of inadequacy, as well as the contextual factors which might maintain such feelings. For example, mental health providers and educators could help perfectionists examine whether they fall short of their own standards or external standards from their family, or both. Additionally, perfectionists could be guided on how to determine whether their own sense of personal inadequacy and distress stems from messages implicitly or explicitly received from their family, especially since both maladaptive individual and family perfectionism appear to be associated with psychological distress (e.g., depression and anxiety). In this regard, Russian SAPS and FAPS may be useful tools for providers and educators working with Russian individuals and students.

The findings also indicate that it is important to consider familial variables in working with Russian students and individuals who struggle with perfectionism. While not all individuals are influenced by their families in the same way, the perception that one’s family has high standards and that there is a discrepancy between their family’s high expectations and their actual performance may have a great impact on distress levels. In other words, problems may arise if individuals perceive that they are not able to attain the high standards others have of them.

Finally, these findings hold implications for how to prevent the negative impact of perfectionism. Russian parents and families who hold high expectations of their children and pressure them to do well could be made more aware of the potential negative impact of placing these expectations on their children. It might be helpful to educate Russian parents and families on the importance of setting healthy and reasonable expectations for their children, as well as teaching them how to support a child who is dissatisfied with their perceived discrepancy between their standards and performance. At the same time, families who work with counselors or educators could be reminded that some types of perfectionism may be beneficial. As seen in the group comparison of perfectionistic categories, the adaptive perfectionistic families still have high standards but low discrepancy. Families can learn to retain the positive benefits of helping their children strive for high achievement, while also teaching them to cope with any perceived gaps between their performance and expectations.

## Conclusion

Although we found evidence of the different types of perfectionistic families in this population and acceptable psychometrics properties of the Russian family perfectionism measure, it is important to note that the sample size was small, of a student population, and imbalanced by gender. Thus, the findings should be interpreted with caution. While our hypothesis that individuals from adaptive and non-perfectionistic families would report less distress as measured by depression symptoms and anxiety was correct, we did not find support for any group differences related to academic performance. However, there was initial evidence that FAPS is a relevant construct for understanding the psychological well-being of perfectionistic college students in Russia. Importantly, the psychometric evaluation of the conducted Russian FAPS suggests that this scale could be used in future research to further explore the relationships between perceived family perfectionism and individual perfectionism among Russian-speaking populations. In fact, the FAPS may support future family interventions and academic evaluations of perfectionism in Russia. Perfectionism is certainly a multidimensional construct with personal and familial considerations, but much more work is needed to better understand how perfectionistic striving can be leveraged within families to enhance the well-being and performance of children and students.

## Limitations

Despite having generated useful findings related to the development of the FAPS and understanding the effects of family perfectionism in Russia, the present study is limited in ways common among survey research. First, the data were cross-sectional, and therefore we are unable to determine causal relationships between family perfectionism and the psychological outcomes measured. Future studies should utilize longitudinal methods to better understand how types of perfectionistic families and individuals predict outcomes related to well-being and academic performance. Relatedly, the sampling (e.g., size and distribution of gender) of the study may have limited the detection and generalizability of effects. Namely, the sample size was limited to 169 participants, which could inhibit detection of small effects. Despite this limitation, the sample size was sufficient for the analyses conducted. However, future research should determine if these findings hold with larger samples. Participants in this study were from a single university in Russia and were predominantly females. Thus, caution is necessary when interpreting the findings, especially relating to generalizing the effects to males or other college students in Russia. Findings from this study may also not be generalized to other populations that might differ in characteristics such as age, geographic location, vocation, etc. Another important limitation is that family perfectionism was measured through self-report from students. Self-reporting of family perfectionism captures merely the student’s perception of family perfectionism rather than the actual levels of family perfectionism or parent’s perception of their imposed perfectionism. Future studies could examine family perfectionism from multiple perspectives and make comparisons on their levels and impact. Finally, the mechanism between family perfectionism, personal perfectionism, and how they impact one’s well-being can be further explored and examined. In addition to the need for longitudinal studies, future studies may also incorporate qualitative methods to gain a more in-depth understanding regarding the interplay among these constructs. Despite the limitations of the current study, the results provide preliminary information regarding the performance of FAPS among this sample, how perceived types of perfectionistic families may vary, and the impact on college student well-being in Russia.
